# Ethics review of studies during public health emergencies - the experience of the WHO ethics review committee during the Ebola virus disease epidemic

**DOI:** 10.1186/s12910-017-0201-1

**Published:** 2017-06-26

**Authors:** Emilie Alirol, Annette C. Kuesel, Maria Magdalena Guraiib, Vânia dela Fuente-Núñez, Abha Saxena, Melba F. Gomes

**Affiliations:** 1 Global Antibiotics Research and Development Partnership (GARDP), Drugs for Neglected Diseases initiative (DNDi), 15 chemin Louis Dunant, 1202 Geneva, Switzerland; 2 World Health Organization, UNICEF/UNDP/World Bank/WHO Special Programme for Research and Training in Tropical Diseases, 20 Avenue Appia, 1211, 27 Geneva, Switzerland; 3 World Health Organization, Department for Information Evidence and Research, 20 Avenue Appia, 1211, 27 Geneva, Switzerland; 4 World Health Organization, 20 Avenue Appia, 1211, 27 Geneva, Switzerland

**Keywords:** Ebola, Research ethics, WHO ethics review committee, Pregnancy, Children, Clinical research, Drugs, Vaccines, Observational studies

## Abstract

**Background:**

Between 2013 and 2016, West Africa experienced the largest ever outbreak of Ebola Virus Disease. In the absence of registered treatments or vaccines to control this lethal disease, the World Health Organization coordinated and supported research to expedite identification of interventions that could control the outbreak and improve future control efforts. Consequently, the World Health Organization Research Ethics Review Committee (WHO-ERC) was heavily involved in reviews and ethics discussions. It reviewed 24 new and 22 amended protocols for research studies including interventional (drug, vaccine) and observational studies.

**WHO-ERC reviews:**

WHO-ERC provided the reviews within on average 6 working days. The WHO-ERC often could not provide immediate approval of protocols for reasons which were not Ebola Virus Disease specific but related to protocol inconsistencies, missing information and complex informed consents. WHO-ERC considerations on Ebola Virus Disease specific issues (benefit-risk assessment, study design, exclusion of pregnant women and children from interventional studies, data and sample sharing, collaborative partnerships including international and local researchers and communities, community engagement and participant information) are presented.

**Conclusions:**

To accelerate study approval in future public health emergencies, we recommend: (1) internally consistent and complete submissions with information documents in language participants are likely to understand, (2) close collaboration between local and international researchers from research inception, (3) generation of template agreements for data and sample sharing and use during the ongoing global consultations on bio-banks, (4) formation of Joint Scientific Advisory and Data Safety Review Committees for all studies linked to a particular intervention or group of interventions, (5) formation of a Joint Ethics Review Committee with representatives of the Ethics Committees of all institutions and countries involved to strengthen reviews through the different perspectives provided without the ‘opportunity costs’ for time to final approval of multiple, independent reviews, (6) direct information exchange between the chairs of advisory, safety review and ethics committees, (7) more Ethics Committee support for investigators than is standard and (8) a global consultation on criteria for inclusion of pregnant women and children in interventional studies for conditions which put them at particularly high risk of mortality or other irreversible adverse outcomes under standard-of-care.

## Background

The Ebola Virus Disease (EVD) epidemic in West Africa began in December 2013 and was declared a “public health emergency of international concern” on 8 August 2014. On 29 March 2016, the World Health Organization (WHO) declared that it no longer constituted such an emergency [1].

Before this epidemic, EVD research was limited to laboratory studies and experience gathered during previous outbreaks [2–9]. Clinical management of EVD cases relied on rehydration, electrolyte replacement, analgesia and treatment of co-infections, with mortality ranging from 40 to 100% depending on age and country [10–12]; no proposed therapy had undergone clinical testing [2, 13, 14]. Vaccines were in very early development [15–21]. A panel of experts convened by WHO in August 2014 concluded that use of unregistered interventions was acceptable provided that laboratory and animal data had yielded positive results [22]. The Panel advised that researchers had “a moral duty to also evaluate these interventions for treatment or prevention, in the best possible clinical trials in order to definitely prove their safety and efficacy or to provide evidence to stop their utilisation.” With the unprecedented scale of the epidemic and the high case-fatality rate, accelerated vaccine and drug development carried the hope that it could help control an epidemic spinning out of control [23, 24].

Clinical trials during emergencies are inherently difficult and raise a number of ethical issues [25–31]. WHO played a prominent role in supporting research. WHO requires all research involving human participants which WHO supports financially or technically to be approved by the WHO Research Ethics Review Committee (WHO-ERC), which was consequently heavily involved in reviews and ethics discussions. This paper describes our experience and reflects lessons for future public health emergencies (PHE).

## Application of WHO-ERC rules of procedure

The WHO-ERC is a 27 member independent committee nominated by the WHO Director-General. The membership is gender and WHO-region balanced, includes expertise in clinical research, drug development, social sciences, legal affairs etc. as well as a lay member. Between 6 and 8 members are from Geneva based universities or international organizations. The WHO-ERC Chair can request input from experts in disciplines not represented on the committee. WHO-ERC members are bound by the rules confidentiality.

The WHO-ERC applies the Council for International Organizations of Medical Sciences guidelines [32]. It normally meets monthly to review protocols meeting the criteria for ‘full committee review’ specified in the WHO-ERC Rules of Procedures (ROP). All submissions require the written assessment and approval of independent scientific reviewers (http://www.who.int/ethics/review-committee/en/).

The ROP provide for ‘accelerated review’ of PHE-related research. The EVD outbreak was the first time accelerated review was implemented. It was facilitated through several measures:

1. An information session was organized to inform all WHO-ERC members about WHO plans for EVD related studies, the drugs and vaccines likely to be evaluated and the context in which studies would be conducted. Documents from related WHO consultations were disseminated [22].
2. A WHO-ERC EVD sub-committee was created to accelerate review of EVD research without compromising other reviews. Members had experience in clinical trials, drug development, infectious diseases and disease surveillance, epidemiology and social sciences and volunteered to review submissions at short notice and attend ad-hoc meetings. No lay person was part of the sub-committee. Other WHO-ERC members were involved in the review to meet expertise or time pressure requirements.
3. Four EVD sub-committee meetings and numerous ad-hoc teleconferences were held in addition to the regular monthly meetings.
4. WHO-ERC members reviewed submissions as received and completed assessment after receipt of missing elements.

## Protocols reviewed

From August 2014 to April 2016, WHO-ERC reviewed 24 new EVD-protocols and 22 amendments. Table 1 and Table 2 show the observational and interventional studies reviewed, including two protocols for ‘Monitored Emergency Use of Unregistered and Experimental Interventions’ (MEURI), a term coined by the WHO Ethics Working Group [33] to distinguish use of unregistered interventions in PHE from compassionate use or expanded access. Four of the five therapeutic intervention protocols did not involve WHO, but WHO-ERC provided its opinion at the request of the chair of the MSF Ethics Review Board [34].

**Table 1:** Observational studies reviewed by the WHO Ethics Review Committee

Study design	Country	Protocol title
Cohort	Sierra Leone	Persistence of Ebola virus in body fluids of Ebola virus disease survivors
Case series	Guinea, Liberia, Sierra Leone	Ebola virus disease due to transmission from survivors: a case series
Case-control	N/A	WHO emergency quality assessment mechanism protocol for laboratory evaluation of Ebola virus antigen-detection in vitro diagnostics
Cross-sectional	Guinea^a^	Etude observationnelle et rétrospective des patients atteints d’Ebola à Conakry, pour déterminer les facteurs de risque de décès
	Guinea^b^	Patients admitted to Ebola treatment centres in Conakry, Guinea: a retrospective observational study
	N/A	Health care providers experiences, values and preferences regarding the selection and use of personal protective equipment in the context of Ebola virus disease outbreaks in Africa
	N/A	A review of the risk factors that contribute to psychological well-being of GOARN and WHO experts involved in Ebola response operation in West Africa
	N/A	Survey of EBOV Exposure and Infection Among Expatriate Aid Workers during the 2014–2015 Ebola Outbreak in West Africa

^a^This study was not approved by the ERC. It was resubmitted as study ^b^and approved by the ERC.

**Table 2:** Interventional protocols reviewed by the WHO Ethics Review Committee

Intervention	Product	R&D Phase	Country	Protocol ID (design)
Vaccine	VSVΔG-ZEBOV	Phase I/II	Switzerland	NCT02287480
			Kenya	NCT02296983
			Gabon	PACTR201411000919191
			Germany	NCT02283099
		Phase II	Guinea (survivors and their contacts and contacts of contacts)	No registration number available.
		Phase III	Guinea (general population)	PACTR201503001057193
			Sierra Leone (general population)	Amendment of PACTR201503001057193
			Guinea (healthcare workers)	Component of PACTR201503001057193
	cAd3-EBOZ	Phase Ib	Mali	NCT002267109
			The Gambia	Not registered, Not initiated.
			Switzerland	NCT02289027
Treatment	Brincidofovir	Phase II	Liberia	NCT02271347
	Favipiravir	Phase II	Guinea	NCT02329054
		MEURI	Guinea	N/A
	MIL 77	MEURI	Sierra Leone, Guinea	N/A
	Convalescent plasma	Phase II/III	Guinea	NCT02342171

## Review times

Table 3 provides summary statistics on review times. WHO-ERC provided the review outcomes within on average 6 working days.

**Table 3:** Review time for EVD-related new submissions

Review type	Days^a^ from complete submission to ERC initial decision Mean, Median (range)	Days^a^ from initial ERC decision to response from WHO RO Mean, Median (range)	Days^a^ from response by WHO RO to final ERC approval Mean, Median (range)	Total Days^a^ from complete submission to final ERC approval Mean, Median (range)
Full-Committee review	6.8, 6 (3–12)	6.8, 7 (0–14)	6.5, 4 (1–18)	18.8, 18 (4–32)
Expedited review^b^	5.4, 4 (0–15)	7.2, 6.5 (2–13)	4.3, 4 (1–9)	14, 17 (0–26)
All types of review	5.8, 5 (0–15)	6.9, 7 (0–14)	5.7, 4 (1–18)	15, 16.5 (0–32)

This table covers only protocols which required ERC review according to WHO-ERC Rules of Procedure (ROP), i.e. protocols of studies for which WHO provides technical support or funding. It does not include protocols reviewed by the ERC at the request of third parties. Only working days are counted, weekends and WHO official holidays are excluded.

Abbreviations: WHO RO, WHO Responsible Officer. As per WHO-ERC ROP, all submissions to WHO-ERC are done by the WHO staff responsible for the study and WHO-ERC feedback is provided to the WHO RO.

^a^Working days

^b^As per WHO-ERC ROP, a protocol is eligible for ‘Expedited Review’ (i.e. review by two WHO-ERC members, but not by the whole committee) if: (i) it will expose research participants to no more than minimal risk i.e. the level of risk to which it will expose participants is no greater than that encountered by people involved in their normal daily activities, such as working at home, in an office, or on a farm, attending school, or undergoing a routine health examination, (ii) minor changes are planned in research that has been previously approved by the ERC and where proposed changes do not create more than minimal risks; (iii) an additional research centre is added to a project previously approved on a multi-centre basis, such that ERC review is limited to ensuring that the necessary local review and approval has taken place.

We minimised time from protocol review to approval by discussion of issues with WHO responsible officers and researchers. The WHO-ERC Secretariat/chair provided hands-on advice for simplifying Participant Information Documents and discussed questions with Data safety monitoring bodies.

## WHO-ERC considerations during protocol review

ECs must evaluate submissions in relation to the specific context of the study and consider how protocol provisions (e.g. interventions, study design, eligibility criteria, community engagement, approach to vulnerable populations) impact autonomy and benefit-risk ratio for participants, justice, scientific validity and social value, and their balance.

Observations that resulted most frequently in requests for justification, clarification or protocol amendment are listed in Table 4. Major challenges and the specific EVD outbreak context are presented below.

**Table 4:** Observations resulting in ERC requests for clarification or amendment of protocols and associated documents

Principle		Observations
Beneficence	Benefit, risk to study participants	• Inconsistencies in participant selection criteria and measures to minimize risk for study participants (duration/type of contraceptives, frequency of follow-up, type of follow up examinations, Data Safety Monitoring Board functioning) between studies examining the same intervention in the same phase of development. • Different standards in the assessment of AEs and SAEs among trials testing the same vaccine. • Unjustified collection of samples.
	Risks to study team or EVD response	• Sub-optimal measures to reduce risk associated with handling of blood samples. • Suboptimal measures to protect Ebola Treatment Units from effect of information about the study and/or the investigational compound.
	Study design - comparator	• Insufficient information on measures to minimize bias in the use of historical controls
	Study design - outcome measures	• Inconsistencies in parameters used to characterize intervention risks • Poor differentiation between deaths due to potential toxicity vs. deaths due to lack of efficacy
	Safety data from prior studies and dose justification	• Lack of information on safety data from previous studies • Lack of documentation on assessment, or insufficient clarity of assessment of safety data by Data Safety Monitoring Board • Lack of documentation of approval of dose by Data Safety Monitoring Board • Insufficient dose justification
Justice	Equitable access	• Lack of justification for exclusion of children and pregnant women • Lack of criteria for trial participant selection when experimental intervention was available in limited quantity
	Impact of studies on routine patient care	• Lack of information on how the study could be conducted in the Ebola Treatment Centres without reducing staff capacity to provide best ‘standard-of-care’ to those not participating in the trial
	Sample and data sharing	• Lack of information on rules and procedures for sample ownership • Lack of information on how results would be shared with participants and their communities
	Future use of remaining samples	• Lack of information on ownership, storage, and disposal of samples. • Lack of information on procedures to determine future use of left-over samples • Lack of information in the Information Documents on future use and shipment of samples.
	Collaborative partnership	• Lack of information on the role of country researchers and health system in study design, planning and implementation
	Accountability	• Lack of information on roles and responsibilities of different investigators
Respect for persons	Information documents	• Information documents provided in technical language with scientific and legal jargon • Inconsistencies between protocol and information document
	Plans for obtaining informed consent	• Study implementation plans with insufficient time planned between informing participants and consent/start of protocol planned procedures • Lack of clarity of criteria to determine potential participants’ ability to provide consent.
	Confidentiality	• Lack of sufficient information on how potential participants other than those in treatment centres or identified during contract tracing were to be approached, and on measures to keep their participation confidential

### Beneficence: Benefits, risks and benefit-risk ratio

Concern for research participants’ welfare is paramount during ethics review. Known and potential risks and benefits of research participation are examined and the benefit-risk ratio assessed [32, 35, 36]. This assessment was complicated by the fact that during the EVD epidemic, conditions and information on Ebola mortality evolved over time and differed between Ebola Treatment Centres (ETC). Mortality figures ranged from 40% to 100% [10–12].

It was important for WHO-ERC that experimental interventions and doses selected had been recommended by an independent scientific committee, a WHO coordinated expert panel (early phase studies) or approved by Data Safety Monitoring Boards (later phase studies). Experimental interventions were evaluated on whether they were likely to have benefit (improve the chances of survival or reduce the probability of infection) or increase risk (result in serious temporary or irreversible adverse reactions, including death). WHO-ERC also evaluated the likely *relative* benefit-risk ratio in the treatment arms proposed, whether participant selection and follow-up plans were suitable for minimizing risks, feasible within the planned setting, could increase inequities in service delivery in the ETC and whether risks to health care workers and response teams were minimized.

For all studies in the EVD-affected countries, WHO-ERC agreed with the benefit-risk ratio assumptions underlying the submitted protocols.

### Beneficence - scientific validity: Study design

Research places participants at known or potential risk with uncertain benefit to the participants for the potential benefit of future patients, i.e. future social value. Studies that are not scientifically valid are inherently unethical, as they fail to meet the social value criterion. Requirements for scientific robustness and social value cannot, however, take precedence over the requirement to maximize benefit and minimize risk for study participants.

Design of interventional EVD trials was controversial and divided opinions between researchers, physicians, ethicists and regulators [26, 37]. At the heart of the controversy was whether randomised controlled trials (RCTs) could (acceptability) or should (moral justification, efficiency, social value) be used to evaluate therapeutics or vaccines for a life threatening disease for which no treatment is available [27, 28, 38, 39]. RCT advocates argued that scientific validity could not be ensured without a comparator arm (i.e. standard-of-care) [28, 30, 37]. Given limited doses of experimental therapeutics and vaccines, RCT advocates also considered that this design best ensured unbiased and fair intervention allocation [37]. On the other hand, many opposed RCTs because of the high case fatality rates under standard-of-care [40]. Adaptive designs were proposed to quickly determine the relative value of different interventions in reducing mortality rates [40]. RCT opponents also argued that the limited number of doses of investigational interventions could preclude adequate RCT sample size, making other designs better suited to provide scientifically valid conclusions [26, 27].

As per WHO-ERC ROP, all protocols had received independent scientific review approval. WHO-ERC considered study design in the context of the combination of the following factors distinguishing the EVD PHE:

- Uncertain epidemic evolution, and hence uncertainty that studies could be completed as planned;
- Limited and/or uncertain availability of investigational interventions;
- Uncertain benefit-risk ratio of investigational interventions and ‘standard-of-care’; the latter differed between ETC and evolved over time, impacting assessment of the relative benefit-risk ratio of the interventions;
- Limited and evolving knowledge about EVD, and uncertainty about effect modifiers/confounders;
- A target population ill-equipped to understand study design implications;
- Limited time for social mobilization and risk posed by communities’ negative perception of research for the EVD response efforts;
- Uncertainty about the requirements for marketing approval of experimental interventions showing favourable results and consequences of this uncertainty for future access to effective and safe interventions.

The therapeutic trials reviewed were single arm non-comparative studies with historical controls (NCT02329054 and NCT02271347) and a comparative, non-randomized study with a concurrent control group who received the standard-of-care when supply of the investigational intervention was insufficient (NCT02342171) (Table 2). WHO-ERC considered these designs appropriate for generating valid scientific knowledge without withholding the intervention from those who could potentially benefit.

For vaccine trials, the decision on whether a placebo is ethically acceptable depends on availability of an established therapy or vaccine, the risk of infection, the associated morbidity and mortality, the location of the study population, and the trial phase. The Phase I/II vaccine trials were dose-ranging placebo controlled, immunogenicity and safety studies in countries not affected by the epidemic (Switzerland, Kenya, Mali, The Gambia, Gabon), with participants randomized between different vaccine doses and placebo [41, 42]. Studies in Switzerland expected some volunteers from organizations which deployed staff to affected countries. WHO-ERC agreed that these studies could allocate volunteers who might be deployed only to vaccine arms to maximize potential for direct benefit.

The Phase III study of the VSVΔG ZEBOV vaccine (PACTR201503001057193) proposed a novel approach to a comparator arm: it randomized contacts of new EVD cases (rings, each constituting a cluster) to immediate or delayed vaccination 21 days after randomization, i.e. no vaccination within the estimated 10–21 days incubation period. The WHO-ERC agreed with the design from the perspective of distributive justice (see below), given immunogenicity data from healthy volunteer studies suggesting a potentially favourable benefit-risk ratio for all vaccinated individuals and considering that (a) participants were at risk of infection, but not infected and thus not at immediate risk of death; and (b) the ‘delayed vaccination arm’ would enhance the social value of the study by providing robust comparative data [43, 44]. WHO-ERC appreciated the additional value of the trial design in providing a ‘herd effect’ to those excluded from vaccination (children, pregnant women) and efficacy assessment under conditions in which the vaccine would be used if found to be safe and effective. There were two main concerns: whether ring members would fully understand the differences in risks between the immediate and delayed vaccination groups and the exclusion of children and pregnant women who were at highest risk of mortality (see below). WHO-ERC asked the team to ensure a strong community-engagement plan to maximize understanding of measures to reduce risk of infection and to facilitate acceptance of exclusion of children and pregnant women.

Observational studies (Table 1) were designed to research the natural history of EVD, specific vulnerabilities of different populations with suspected or confirmed EVD and virus persistence in survivors [45, 46]. WHO-ERC considered all suitable for yielding valid information for guidelines and prevention strategies in future Ebola epidemics. The most challenging issue for WHO-ERC was how and when negative test results ought to be communicated to participants in the virus persistence studies given uncertain diagnostic test performance. WHO-ERC favoured informing participants after each test with appropriate precautions, balancing this against the risk of loss of trust and potential spread of infection engendered by non-communication. To improve understanding of test limitations and lessen the risk of stigmatization for survivors with positive EVD-test results, WHO-ERC requested piloting the information documents with survivors and intensive training of counsellors providing the test results.

### Justice - exclusion of children and pregnant women

The ethical principles of justice (fairness, equity and maximisation of benefit) are often undermined by exclusion of children and pregnant women from clinical research. A large proportion of medicines are used in children and pregnancy without sufficient evidence in these populations, including on the appropriate dose [47–50].

EVD-related maternal mortality was around 90% in previous outbreaks, foetal and newborn loss approached 100%, and mortality rates were highest among children ≤4 years [10, 51–54]. Consequently, exclusion of pregnant women and children from clinical trials denied the potential benefits of interventions to those most severely affected. Furthermore, lack of trial data would translate into their exclusion from registration and hinder future access to effective interventions.

WHO-ERC systematically requested protocol amendments to include pregnant women and children (including unaccompanied minors), unless their exclusion was justified by data demonstrating that the risk of treatment was likely to exceed risks with ‘standard of care’. Given the time pressure for study initiation and mindful that legal issues might be driving exclusion of these groups, the trade-off was between ‘delayed initiation with potential benefit for all’ versus ‘immediate initiation with potential benefit for the majority’ and WHO-ERC did not insist on protocol amendments when data-based justifications were not provided.

### Justice - data and sample sharing

In 13/24 (54%) protocols the investigators proposed obtaining biological samples and in 33% (8/24) retaining unused samples for future use. Such samples provided an unique opportunity for creating a common good and contributing to development of future EVD diagnostics and interventions. Discussions on an international bio-bank with global governance structure started early during the epidemic but such a bio-bank was not in place when WHO-ERC reviewed protocols [55, 56].

WHO-ERC requested clarifications of sample and data ownership, data sharing policy, processes for decisions on future use of samples and appropriate participant information. In view of the urgency, WHO-ERC approved studies based on researcher commitment to put appropriate agreements/processes in place.

### Respect for persons - informed consent, collaborative partnership, community engagement and monitoring of study implementation

Therapeutic trials took place in ETCs where potential participants were likely to be extremely sick, isolated, and informed about the research by staff wearing protective equipment with limited time for presenting and discussing each study. Patients were probably aware of their risk of death and likely to perceive study participation as their only chance for survival. WHO-ERC was mindful of the limitations of obtaining *informed voluntary* consent in this context and suggested ways of easing information and consent procedures. Simplifying language and reducing information document elements, encouraging dialogue while EVD diagnosis was being confirmed and before isolation in ETCs were approaches proposed to increase understanding and reduce the risk of ‘situational coercion’.

For two protocols, consent to retrieve anonymized information from patient records was waived because data would improve understanding of the disease (high social value) and seeking consent from previous, sometimes deceased patients, would have been impractical.

The social environment was characterized by distrust of large fractions of the populations towards many involved in responding to the epidemic, especially those from the international communities. Consequently, WHO-ERC considered involvement of local scientists familiar with culture, attitudes, language and socio-economic context even more important than for other studies and indispensable despite the strain of the outbreak on local resources. In at least 8/24 protocols reviewed, the role of local stakeholders was insufficiently specified and WHO-ERC sought to obtain clarification of their involvement.

Good Clinical Practice Guidelines require sponsors to verify study implementation per protocol through monitoring [57, 58]. WHO-ERC reviewed monitoring plans of all prospective studies, realizing that standard monitoring approaches were not feasible for studies in ETCs. In view of the enrolment of individuals at risk of, or survivors of, EVD, WHO-ERC reviewed monitoring reports with particular attention to implementation of measures to improve understanding of procedures, risks and test results (see above). In one instance WHO-ERC requested an additional independent monitoring visit.

## Recommendations for future outbreaks/PHEs

The 2013–2016 Ebola outbreak, the largest ever, was marked by high mortality and high levels of uncertainty. Information normally well-defined in research protocols – standards of care, levels of risks, age and gender-specific morbidity and mortality rates – was uncertain, changing and different between ETCs. Everyone, including front-line workers, researchers and Ethics Committees (ECs) worked with imperfect and uncertain data under pressure [10, 11, 34, 40, 45, 59].

Other outbreaks/PHE will have different epidemiology, treatment options and level of uncertainty and will occur in different health care, economic and socio-psychological settings. Because ECs must always evaluate submissions in relation to the specific context, our recommendations can only address the common characteristics of outbreaks/PHEs.

The hallmark of a PHE is the urgency to control and to conduct research in the face of uncertainty, suboptimal conditions and pressure. The tension between obtaining knowledge for the benefit of future patients and the interests of participants is heightened during outbreaks/PHEs, in particular when the risk of irreversible morbidity and/or mortality is high, there are many unknowns and research to identify interventions is urgent.

EC approval is the last step before research implementation and the prospect of achieving some good. Our experience leads us to propose measures to facilitate EC approval that we consider applicable to any outbreak/PHE (as well as outside PHEs). The Ethics Review Board of Médecins Sans Frontières and a ‘Committee on Clinical Trials During the 2014–2015 Ebola Outbreak’ constituted by the US National Academy of Science to analyse the EVD clinical trials make some similar and complementary recommendations [34, 60].

## Preparation of submissions

Most issues that prevented immediate approval (Table 4) were avoidable and not EVD-specific: (i) insufficient or inconsistent information on key elements (e.g. sample size, inclusion-exclusion criteria, safety follow-up, community sensitization, role of local researchers); (ii) lengthy, technical participant information documents inappropriate for the target population; (iii) missing information on provisions for sharing and use of data, governance and jurisdiction over leftover samples, study coordination and governance.

We recommend that investigators invest time in ensuring submissions are internally consistent and complete, with information documents participants can understand (e.g. through pre-submission quality control by somebody not involved in document preparation but familiar with ethics guidelines and templates/check-lists many Ethics Committees provide and by testing information document comprehension with representatives of the target population).

## Improved collaboration between local and international researchers

Given the resource-limited settings in the EVD-affected countries, research studies were often led by international staff and the roles of local researchers frequently unclear (see above, Table 4). Research participants were invariably seriously ill, at risk of infection, or facing stigmatization and its consequences, afraid, vulnerable, and unclear about the objectives, benefits and risks of study participation and the distinction between research and medical practice. Many of these characteristics will be encountered in other PHEs. For community and participant engagement before, during and after a study based on understanding of the local culture, attitudes, and socio-psychological situation, and appropriate study implementation plans, close collaboration of local and international researchers is essential.

## Template agreements for data and bio specimen ownership and use

Information on sample and data ownership, data sharing policy, processes for determining future use of samples was frequently insufficient (see above, Table 4). Related agreements are critical for ensuring study participants and communities (in the widest sense) benefit from study results. Negotiating agreements is complex and consumes time not available in outbreaks/PHE. Template agreements emerging from international consultations, e.g. the discussions about an international Ebola bio-bank [55, 56] could accelerate study-specific agreements on data and bio-specimen ownership, governance of bio-specimen use and affordable access to any resulting licensed interventions or diagnostics.

## Scientific, data and safety monitoring, and ethical review

In some cases, scientific review and data safety monitoring committee reports did not reassure WHO-ERC that all relevant data had been considered and WHO-ERC required confirmation or access to these data which delayed feedback.

We recommend creation of a Joint Scientific Advisory Committee and a Joint Data Safety Monitoring Committee for studies linked to a particular intervention, or group of interventions (e.g. the same vaccine) to ensure (and reassure ECs) that recommendations for one study (e.g. dose selection) are made with detailed knowledge of the results of all other relevant studies. The ethical value of cross-trial data sharing between sponsors and Data Safety Review Committees has previously been suggested previously [61].

Many trials required approval from several ECs. Although many EC reviews were consistent, some were discordant [34, 62]. Consolidating reviews was explored but proved largely unfeasible. We recommend the constitution of a Joint Ethics Review Committee (J-ERC) with representatives of the ECs of all relevant countries/institutions [63]. Their secretariats would decide on distribution of secretarial functions. A J-ERC might face practical and legal issues, but the advantages should motivate efforts to overcome these: A J-ERC could strengthen reviews because members would represent the range of perspectives without the ‘opportunity cost’ of multiple reviews delaying final approval. Furthermore, a J-ERC would be more aware of potentially ‘competing’ studies planned in the same country or centre and could address resulting ethical issues (e.g. implications for potential participants of simultaneous studies at the same site with the same or very similar eligibility criteria). A J-ERC might feel more comfortable to modify/waive informed consent requirements when truly *informed voluntary* consent is unlikely. J-ERC members might be authorised to engage closer with researchers than is standard to minimize time to approval of study plans/information documents (e.g. discussion of revised information documents before re-submission). Direct communication between the Chairs of the J-ERC, the Joint Scientific Advisory and the Joint Data Safety Monitoring Committees could further reduce time to approvals.

## Review of criteria for inclusion of pregnant women and children

Inclusion of women in clinical research was achieved in the 1990ies [64–66] but pregnant women continue to be excluded, even from Phase IV studies on conditions affecting them [67]. In the US, all medicines for non-obstetrical conditions in pregnant women have to be used ‘off-label’ [65, 68]. Pregnant women and treating physicians thus face the choice between the risks of no treatment and the risks of ‘de-facto clinical research’ without the rigour and safeguards of prospective clinical trials. Pregnancy registries collecting outcomes from such ‘de-facto clinical research’ are currently the primary source of information on the effects of drugs taken by women during pregnancy and lactation [69]. Lack of clinical trial data to inform treatment of children has motivated regulatory requirements and incentives for such trials [47–50, 70].

Despite the high EVD-related mortality among children, pregnant women/foetuses and neonates [10, 51–54], exclusion of pregnant women and children in EVD research protocols was a consistent and intractable problem. Denial of the potential benefits of trial participation to pregnant women, foetuses and children resulted in *individual injustice*. Their exclusion as groups resulted in *social injustice* and reduced social value. This will translate into less, if any, availability of the interventions for pregnant women and children during the next epidemic, further reducing *social justice* and value of the trials. The EVD epidemic thus exposed standard clinical trial approaches in drug and vaccine development that missed the opportunity of including these groups in clinical research.

Among the challenges faced, we consider this the most difficult to address since it involves scientific, ethical, and also legal liability issues. A global consultation on inclusion of children and pregnant women in trials for conditions putting them and the foetus/new born at high risk of mortality or other irreversible adverse outcomes should be convened to increase *individual* and *social justice* of future trials and increase potential for changes to guidelines, regulations and future practice.

## Conclusions

Despite time and psychological pressure, we did not identify shortcuts to fulfilling our mandate conscientiously; the moral and ethical imperative was to examine the submissions as thoroughly and with the same type of considerations the WHO-ERC applies to studies outside PHE. Future outbreaks/PHEs will differ from the EVD outbreak in epidemiology, morbidity and mortality, treatment options, health care system capacity and psycho-socio-economic context which affects the ethical acceptability of protocols. Our experience suggests that advance agreements on the principles governing bio-specimen and data use, prior consultations on inclusion/exclusion of pregnant women and children, and exploration of joint scientific, data safety monitoring and ethical reviews early in the next PHE could accelerate finalization of study plans and ethics approval; in other respects there is no substitute for good research protocols and thorough EC review, even under pressure.

## Abbreviations

EC: Ethics Committee
ETC: Ebola Treatment Centres
EVD: Ebola Virus Disease
J-ERC: Joint Ethics Review Committee
MEURI: Monitored Emergency Use of Unregistered and Experimental Interventions
PHE: Public health emergencies
RCT: Randomized controlled trial
ROP: Rules of Procedures
WHO: World Health Organization
WHO-ERC ERC: World Health Organization Ethics Review Committee
