# Erratum to: Ethics review of studies during public health emergencies - the experience of the WHO ethics review committee during the Ebola virus disease epidemic

**DOI:** 10.1186/s12910-017-0204-y

**Published:** 2017-07-12

**Authors:** Emilie Alirol, Annette C. Kuesel, Maria Magdalena Guraiib, Vânia de la Fuente-Núñez, Abha Saxena, Melba F. Gomes

**Affiliations:** 1 Global Antibiotics Research and Development Partnership (GARDP), Drugs for Neglected Diseases initiative (DNDi), 15 chemin Louis Dunant, 1202 Geneva, Switzerland; 2 World Health Organization, UNICEF/UNDP/World Bank/ WHO Special Programme for Research and Training in Tropical Diseases, 20 Avenue Appia, 1211, 27, Geneva, Switzerland; 3 World Health Organization, Department for Information Evidence and Research, 20 Avenue Appia, 1211, 27, Geneva, Switzerland; 4 World Health Organization, 20 Avenue Appia, 1211, 27, Geneva, Switzerland

## Erratum

After publication of the article [1], it has been brought to our attention that an author’s name has been included incorrectly. The correct name is “Vânia de la Fuente-Núñez”. The original version of the article has been revised to reflect this.
